# A Case of Twisted Ovarian Dermoid Cyst During Pregnancy

**DOI:** 10.7759/cureus.33582

**Published:** 2023-01-10

**Authors:** Anupama V Dhobale, Mangesh G Kohale, Sagar V Jha, Nandkishor J Bankar, Pratima Khatake

**Affiliations:** 1 Obstetrics and Gynaecology, Datta Meghe Medical College, Datta Meghe Institute of Medical Sciences (DU), Wardha, IND; 2 Pathology, Datta Meghe Medical College, Datta Meghe Institute of Medical Sciences (DU), Wardha, IND; 3 Obstetrics and Gynecology, Datta Meghe Medical College Nagpur, Datta Meghe Institute of Medical Sciences (DU), Wardha, IND; 4 Microbiology, Jawaharlal Nehru Medical College, Datta Meghe Institute of Medical Sciences (DU), Wardha, IND; 5 Physiology, Government Medical College Baramati, Pune, IND

**Keywords:** cystic teratoma, sebaceous, adnexal, laprotomy, ectopic pregnancy, ovarian cyst torsion, dermoid cyst

## Abstract

Ovarian cysts are common during pregnancy as an outcome of routine prenatal ultrasounds. Although most cases are benign, complications, such as torsion, rupture, and malignant changes, can occur. Torsion risk increases fivefold during pregnancy. It is extremely hazardous to expectant mothers and unborn children. In a rural health tertiary center, we report the case of a 23-year-old primigravida with 14 weeks of pregnancy presented with acute abdomen and nausea, vomiting for four hours. On ultrasonography, she was diagnosed with a 14 cm × 11 cm left dermoid cyst. She underwent a laprotomy, and a twisted dermoid cyst was found; therefore, a left oophorectomy was performed with consent. Histopathological examination revealed the presence of a dermoid cyst. She is regularly followed up at our center with a healthy intrauterine fetus growing within. Although antepartum surgical intervention has been proven safe, there are some risks associated with abdominal surgery for both pregnant women and their unborn children. As a result, the management strategy must be chosen based on a risk-benefit analysis of adnexal mass characterization and gestational age.

## Introduction

Dermoid cysts, or mature cystic teratomas, are the most common ovarian tumors among women of reproductive age, making up a remarkable 20% of all adult ovarian tumors [1,2]. These structures stem from germ cells and usually comprise endodermic, ectodermal (e.g., skin, hair, and nails), and mesodermal (e.g., fat and muscle) tissues [3]. Approximately 0.3% of pregnancies contain a dermoid cyst, which is usually detected in the second trimester [4]. During pregnancy, dermoid cysts are more likely to cause complications such as torsion, rupture, and infection [5]. This case report details an incident of a huge ovarian dermoid cyst twisting during the second trimester of gestation and its subsequent treatment approach.

Adnexal torsion around the vascular axis or pedicle is referred to as ovarian torsion. Common risk factors include a free mobile cyst with a long pedicle. Acute abdominal pain during pregnancy can exhibit nonspecific signs and symptoms related to various diseases. The infundibulopelvic and tubo-ovarian ligaments form a central line axis, and when the adnexa, ovary, or fallopian tube rotate completely around this axis, it sparks an event known as adnexal torsion [6]. This leads to a decrease in venous flow, which a decline may follow in ovarian arterial flow. Hemorrhage, infarction, necrosis, and stromal edema are all caused by decreased blood flow [7]. This condition makes up around 3% of all gynecological surgical crises among women, with 80% of them developing within the childbearing age [8]. Ovarian torsion during pregnancy is uncommon, occurring in only one in every 5,000 pregnancies [9].

## Case presentation

A 23-year-old primigravida presented to the ANC clinic with severe, non-radiating, unrelieved abdominal pain and four episodes of vomiting for four hours. Her symptoms were severe and unrelieved by medication, and she had no history of vaginal discharge or bleeding. The patient conceived naturally without treatment and provided no information about any illnesses, fevers, urinary problems, diarrhea, or constipation. The patient had one visit at eight weeks of gestation and had no relevant medical or surgical history. On general examination, her blood pressure was 90/60 mmHg, pulse rate was 112/min, afebrile, and her cardiovascular and respiratory systems were normal. A tender adnexal cystic mass was detected on abdominal examination, in addition to a 14-week palpable uterus. These findings were corroborated by vaginal examination.

All of her blood and urine test results were within normal limits. Ultrasonography revealed a 14 cm × 11 cm single mass lesion in the left iliac fossa with thin-walled septa and dermoid cyst components. It also showed a single intrauterine live fetus of 14 weeks with a placenta developing in the fundoposterior segment.

**Figure 1:**
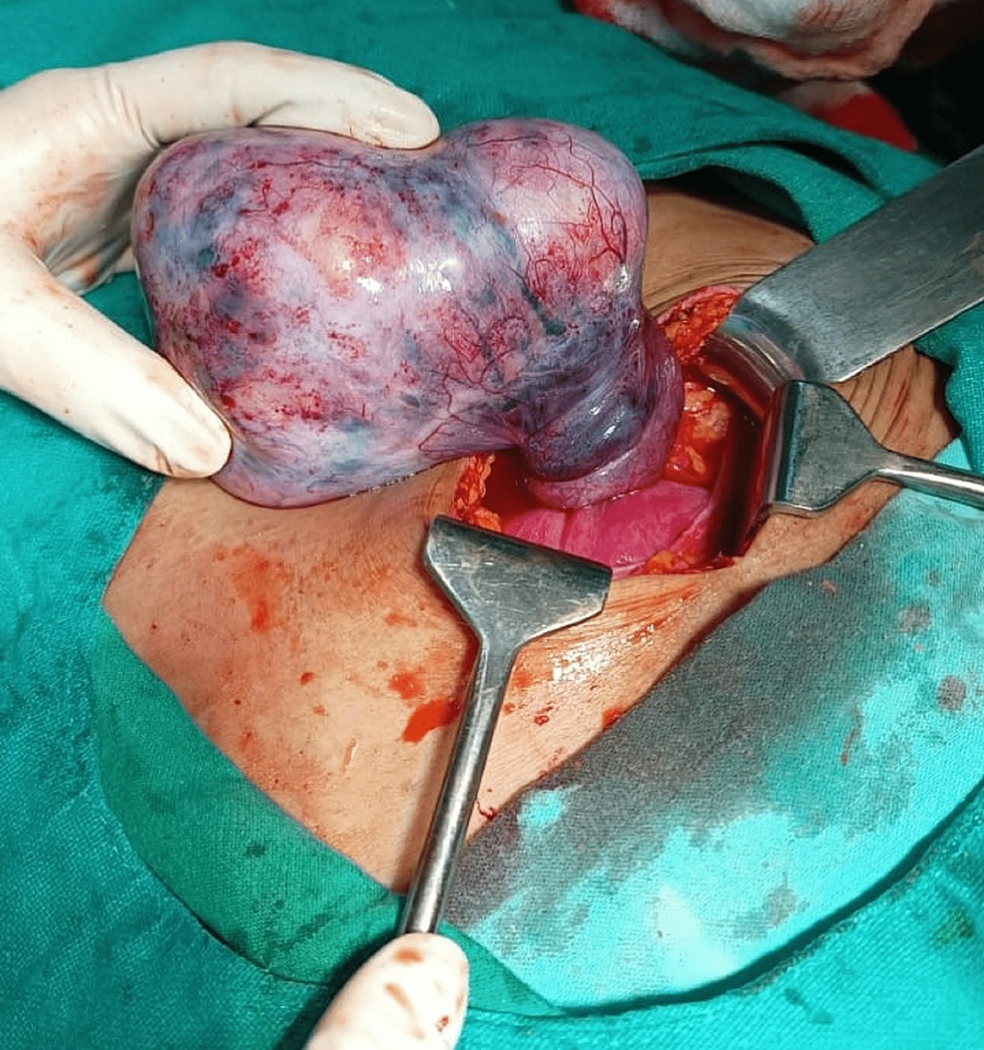
Twisted dermoid cyst

**Figure 2:**
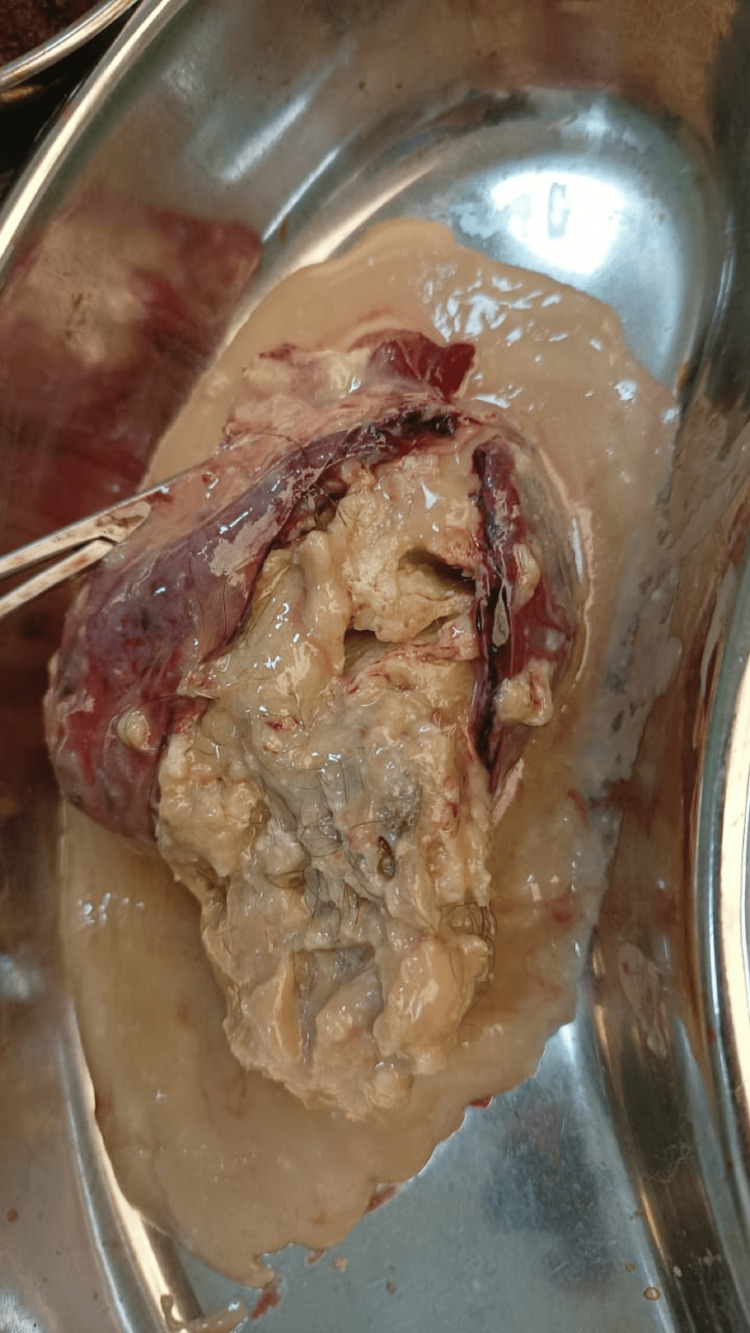
Contents of dermoid cyst

 With proper consent and in view of an acute emergency patient was posted for emergency laparotomy under spinal anesthesia after giving a stat dose of intramuscular synthetic hydroxy progesterone, 500 mg. A right ovarian cyst measuring 14 cm x 10 cm was visualized to be triply rotated around its pedicle, as shown in Figure 1. Oophorectomy was done immediately after untwisting it. A histopathological examination was requested for the cyst, contents are shown in Figure 2. The other ovary was found to be normal. The procedure was uneventful.

The patient was kept on Intravenous tocolytics for first^ ^postoperative day followed by oral tocolytics 10 mg twice a day for 14 days. A normal postoperative obstetric ultrasound suggesting live 15 weeks fetus was observed on the ninth postoperative day when the patient was discharged. Being a vertical scar and far-stay, the patient was willing to stay till suture removal. The patient is being monitored regularly and the fetus is healthy and growing well. The resected dermoid cyst of the ovary was diagnosed on the basis of the histopathological examination showing keratinizing, stratified squamous epithelial lining, intraluminal keratin, hair and sebaceous glands.

## Discussion

During pregnancy, the most common ovarian tumors are serous cystadenomas, luteomas, corpus luteal cysts, and cystic teratomas [10]. An appendix abscess, diverticular abscess, pelvic kidney, ectopic pregnancy, retroperitoneal tumors, a non-pregnant uterine bicornuate horn uterus, and a retroverted gravid uterus may all be alternative diagnoses [10]. Ovarian torsion occurs most commonly in the first trimester and is least common during the third trimester [8]. Cysts less than 6 cm in diameter are usually not treated aggressively unless they appear malignant on ultrasound; in most cases, they may resolve spontaneously. Simple cystic appearances may be dealt with expectantly using serial ultrasound. Cysts > 10 cm in diameter are often removed due to an increased risk of malignancy, rupture, and torsion. If the cyst contains septa, papillary excrescences, nodules, or solid matter, it should be resected. On USG, dermoid cysts may exhibit widespread or partial echogenic masses, with echogenic areas containing sebaceous material and hair. Although emergency exploratory laparotomy may be necessary to treat rupture, twisting, or infarction. In as many as 50% of cases, the management of pregnant patients has improved because of imaging procedures, such as high-definition ultrasound, MRI, and transvaginal color Doppler. Regardless of the stage of pregnancy, an ovarian cyst that ruptures, twists, or exhibits signs of cancer must be surgically removed [11]. To identify the issue, a strategy involving multiple imaging techniques was necessary. Ultrasonography and MRI tests were attempted first, but they were not able to give a definitive answer. So, a CT scan was used next, which ultimately provided the necessary information. All-in-all, it is suggested that a combined imaging technique should be used [12]. A pregnant woman presenting with acute abdomen with a provisional diagnosis of twisted ovarian mass of more than 10 cm, we performed the indicated surgical treatment to reduce the risk of complications in both the mother and fetus.

## Conclusions

Ovarian torsion during pregnancy is rare and relatively little is known about the epidemiology of ovarian torsions, since only 1%-2% of pregnant women present with this condition, and most cases go undetected. Although most cases are asymptomatic, a large cyst can cause significant maternal and fetal morbidity rates if it ruptures or twists. Dermoid cysts are usually benign but may be involved with malignancy. To conclude, obstetricians should be alert to the possibility of acute ovarian torsion in expecting mothers and should be highly suspicious of it. Prompt surgical action benefits both the mother and the unborn baby.
